# Measuring participants’ immersion in healthcare simulation: the development of an instrument

**DOI:** 10.1186/s41077-016-0018-x

**Published:** 2016-05-23

**Authors:** Magnus Andersson Hagiwara, Per Backlund, Hanna Maurin Söderholm, Lars Lundberg, Mikael Lebram, Henrik Engström

**Affiliations:** 1 Centre for Prehospital Research, Faculty of Caring Science, Work Life and Social Welfare, University of Borås, 501 90 Borås, Sweden; 2 School of Informatics, University of Skövde, Box 408, 541 28 Skövde, Sweden; 3 Centre for Prehospital Research, Swedish School of Library and Information Science, Faculty of Librarianship, Information, Education and IT, University of Borås, 501 90 Borås, Sweden; 4 Swedish Armed Forces Centre for Defence Medicine, Box 5155, 426 05 Västra Frölunda, Sweden

**Keywords:** Healthcare simulation, Immersion, Measure, Instrument

## Abstract

**Background:**

Immersion is important for simulation-based education; however, questionnaire-based instruments to measure immersion have some limitations. The aim of the present work is to develop a new instrument to measure immersion among participants in healthcare simulation scenarios.

**Methods:**

The instrument was developed in four phases: trigger identification, content validity scores, inter-rater reliability analysis and comparison with an existing immersion measure instrument. A modified Delphi process was used to develop the instrument and to establish validity and reliability. The expert panel consisted of 10 researchers. All the researchers in the team had previous experience of simulation in the health and/or fire and rescue services as researchers and/or educators and simulation designers. To identify triggers, the panel members independently screened video recordings from simulation scenarios. Here, a trigger is an event in a simulation that is considered a sign of reduced or enhanced immersion among simulation participants.

**Results:**

The result consists of the Immersion Score Rating Instrument (ISRI). It contains 10 triggers, of which seven indicate reduced and three enhanced immersion. When using ISRI, a rater identifies trigger occurrences and assigns them strength between 1 and 3. The content validity analysis shows that all the 10 triggers meet an acceptable content validity index for items (I-CVI) standard. The inter-rater reliability (IRR) among raters was assessed using a two-way mixed, consistency, average-measures intra-class correlation (ICC). The ICC for the difference between weighted positive and negative triggers was 0.92, which indicates that the raters are in agreement. Comparison with results from an immersion questionnaire mirrors the ISRI results.

**Conclusions:**

In conclusion, we present a novel and non-intrusive instrument for identifying and rating the level of immersion among participants in healthcare simulation scenarios.

**Electronic supplementary material:**

The online version of this article (doi:10.1186/s41077-016-0018-x) contains supplementary material, which is available to authorized users.

## Background

Manikin simulation has become an established educational method in healthcare education and training [1], enabling a learner-centred and patient-safe role-playing approach to training where the trainees experience different scenarios. The ability to imagine these possible future situations is a key factor in this type of training [2]. This ability, in combination with the properties of the simulation, is important for simulation trainees’ acceptance of the training situation as being believable and adequate for its purpose. In our case, this means that the trainee must believe that the consequences of the actions taken are represented as if they would occur in a real situation, even though they are not.

There are a number of concepts which relate to the above experience of involvement, such as *immersion* [3, 4], *flow* [5], *presence* [6], *cognitive absorption* [7], *buy-in* [8], *suspension of disbelief* [9], *fiction contract* [2] and the *as-if* concept [2]. According to Dieckmann et al. [2], the *as-if concept* is a “corner stone of effective simulation” (p.188) illustrated by an illuminating example on p.189: “*Consider an anaphylaxis case and how easily participants often can and do integrate a verbal description of a rash on the patient’s chest that they cannot actually see. They often act as if the rash would be given. However, we still do not know enough about the conditions under which they do so or not*.” The as-if concept has similarities with immersion in that it relates to a subjective participant experience of a simulation and a suspension of disbelief, but there are no reported efforts of using the as-if concept to study participant experience. Immersion covers a wide range of experiences, and the concept has been used in a number of different application areas [4, 10, 11]. An additional strength of the immersion concept in this context is that it has been used by Jennett et al. [12] to develop a validated instrument to measure participant engagement. In this paper, we focus on immersion as defined by Dede [4]: “*Immersion is the subjective impression that one is participating in a comprehensive, realistic experience*” (p.66). We argue that immersion is a good candidate to describe and understand participants’ experiences during simulator training in healthcare education. However, subjective experiences and emotions are complex, and there is an ongoing debate about to what extent they are objectively measurable [13]. Even though immersion is defined as a subjective impression, we argue that it is constructed and externalized in participants’ interactions with artefacts, simulators, instructors and other participants and that those interactions can be observed.

There is a limited amount of studies that have investigated the effect of immersion on learning. For example, in digital environments immersion has been suggested to enhance education in three ways [4]. First, immersion can assist the learner to apply multiple perspectives. Second, immersion is important for situated learning which is considered a powerful pedagogy, and third, immersion can improve transfer of knowledge to the real world. There are, however, threats related to immersive aspects of training. Based on research of war gaming in military training, Frank [14] proposes the concept *gamer mode* which means that a trainee switches into a totally immersed mode focusing on winning the game at the expense of the intended learning. Irrespective of whether the relation between immersion and learning is positive or negative, there is an apparent need to better understand immersion in training contexts.

Today, the primary approach to measure immersion is through questionnaires, capturing participant-based, perceived immersion. A number of different questionnaires where the participants of a simulation report their immersion tendencies and perceptions of the simulation exist, one prominent being the immersion questionnaire presented by Jennett et al. [12]. Often, these are developed for virtual and/or highly technological environments. Furthermore, as discussed by Wallis & Tichon [15] when investigating immersion, presence and learning, questionnaires might have limitations in terms of evaluative power when used in experiments. Another limitation of immersion questionnaires [12] (and all questionnaire-based instruments) is that they are intrusive and require the subject to articulate their experience. Efforts have been done to complement or combine questionnaires with psychometric measures such as EEG, skin conductance, pulse and blood pressure [16–18]. The strength of psychometric instruments is that they do not require subjects to explicitly articulate their responses; they are, however, intrusive in that they typically require subjects to wear sensors. Hence, richer, structured and non-intrusive evaluation approaches are needed to better analyse immersion in healthcare simulation.

The aim of this paper is to present the Immersion Score Rating Instrument (ISRI) and how it was developed and validated. ISRI allows observers as educators and researchers to analyse training sessions to observe situations where the participants externalize behaviour that may be related to their immersion in the scenario. By observing these *triggers* related to immersion, we aim to better understand how the trainees experience the training scenario. Triggers are defined as indicators that point to *reduced immersion* (e.g. just verbalizing/saying that you deliver a treatment rather than actually doing so) or *enhanced immersion* (e.g. actually trying to calm the simulator by comforting it). The observed changes in behaviour form indications of being in or out of immersion. When using ISRI, observers assign strengths to each identified trigger, which are used to calculate an immersion score similar to that of Jennett et al. [12]. In addition to being non-intrusive, ISRI allows for deeper analysis and exploration of factors that affect immersion. The triggers may be seen as observable signs of reduced or enhanced immersion. This can add valuable information as a complement to the participants’ self-reported experiences, or on its own when self-reported measures such as questionnaires are infeasible or obtrusive. However, it should be noted that this is not to say that these observations are objective measurements of the participants’ subjective experiences.

## Methods

The ISRI development process was based on video recordings of healthcare simulation scenarios and performed in four phases: (1) trigger identification, (2) content validity analysis, (3) inter-rater reliability analysis and (4) comparison between ISRI and postquestionnaire outcomes.

### Sample and setting

A sample of educators/researchers in prehospital care, nursing, medical simulation, emergency medicine, information science and serious gaming were recruited to a Delphi panel (Table 1). The sampling of the Delphi panel was purposively selected, that is, the participants were selected based on their professional knowledge and experience of the topic (immersion and healthcare simulation). The panel members were recruited from a school of informatics with focus on serious games, one centre for prehospital research and a centre for defence medicine. All the members in the panel had previous experience of simulation in the health services and/or fire and rescue services as researchers and/or educators and simulation designers, e.g. [19–21].

**Table 1:** Panel characteristics

Employment role	Number
Researcher in serious gaming	5
Researcher/educator in nursing and prehospital care	3
Researcher/educator in medicine, medical simulation and emergency medicine	1
Researcher in information science	1
Panel members (total)	10

The video recordings that served as a basis for the instrument development process were recorded during a healthcare simulation experiment in November 2014. In the experiment, 12 professional ambulance teams (24 ambulance nurses) from four different ambulance organizations were recruited. All the participants were working full time as ambulance nurses and had earlier experience with simulation. The 12 ambulance teams participated in two simulation scenarios: one *basic*, mirroring how simulator training currently is done in the region where the experiment was done, and one *contextualized*, where we strived to capture more of the complexity of the work process. In the basic simulation scenario, the participants assessed and treated a simulator manikin in a regular lecture room setting. In the contextualized scenario, the whole chain of an ambulance mission was represented (including dispatch, driving, on-scene treatment, transport and handover at hospital) and extra efforts were made to create realistic environments. The contextualized scenarios can be labelled as in situ simulation. The scenarios were organized in blocks in order to vary: (1) the *type* of medical scenario (“elderly man with respiratory distress” or “drug addict with respiratory distress”) in each of the conditions (basic/contextualized) and (2) the *order* in which participants did the scenarios. Informed consent (oral and written) was obtained from the participants during an introduction to the experiment. The study was approved by the research ethics adviser at the University of Borås and conducted in accordance with the ethical recommendations of the Swedish Research Council [22]. A detailed description of the experiment and scenario design can be found elsewhere [23].

#### Phase 1: trigger identification

The first phase in the instrument development process was conducted as a modified Delphi process [24] with the aim to identify triggers. To do this, we used video recordings of prehospital personnel participating in a simulation-training scenario (described above). As an example, a sequence in the video recording where the ambulance nurses interacted with the manikin as if it was a real person could be considered as a trigger indicating enhanced immersion. Sequences where the prehospital personnel ask the simulation instructor a question could be a trigger indicating reduced immersion.

*In the first round*, all the panel members (10 in total) received two different video recordings from the total 24 videos from the experiment, one basic and one contextual, to examine. Altogether the panel examined 20 video records [23]. The panel members independently screened the video recordings and were instructed to identify triggers. Next, the identified triggers were sorted into a matrix, preserving the original expression of the panel member as much as possible [24]. In the next stage, the identified triggers were clustered in order to categorize the events and reduce the categories to a manageable amount.

*In the second round*, the clusters were sent back to the panel for discussions. Panel members were asked to classify the triggers as important or not important. They were also asked to add potential new triggers to the list.

*In the third round*, the panel members received the updated trigger list where less important triggers were deleted and new triggers added and were asked to classify the triggers as enhancers or reducers of immersion. They also scored the importance of each trigger on a Likert scale: 1 = not important, very unlikely that this trigger is a sign of enhanced or reduced immersion; 2 = not very important; 3 = possibly important, possible a significant trigger; 4 = important, in most instances, this trigger is a sign of enhanced or reduced immersion; and 5 = extremely important, very likely that this trigger is a sign of enhanced or reduced immersion. If at least 80 % of the panel members rated a trigger as 4 or 5, it was considered as an important trigger and qualified for the next round.

The panel members were asked to add new triggers and describe them with a few words. They were also asked to assess the phrasing of all the triggers.

*In the fourth round*, the process in round 3 was repeated using the new phrasings. If any of the new triggers were to be rated by at least 80 % of the panel members as 4 or 5, they would be added to the trigger list.

#### Phase 2: content validity analysis

In order to ensure criterion-related and construct validity of instrument items (here triggers), Polit and Beck [25] recommend a content validity analysis to be conducted. Panel members were asked to individually grade the relevance of the 10 triggers remaining after clustering and reduction, using a 4-point rating scale: 1 = not relevant, 2 = somewhat relevant, 3 = quite relevant and 4 = highly relevant. The panel members’ ratings were used to calculate the content validity index for items (I-CVI) and content validity index for scale (S-CVI). For the I-CVI calculation, the number of panel members rating an item as either 3 or 4 was divided by the total number of panel members. Triggers with an I-CVI score larger than 0.78 were considered content valid [25]. The S-CVI score was calculated by adding together the I-CVI score for all the triggers and divide the sum by the number of triggers. The result is the S-CVI/Ave score. An S-CVI/Ave of 0.90 or higher is considered as high-scale-level content validity [25].

#### Phase 3: inter-rater reliability analysis

The inter-rater reliability (IRR) analysis was performed on the final ISRI instrument by six raters independently rating the same video-recorded scenario from the experiment sample [23]. An inter-rater training session was performed before the IRR analysis. The training session lasted 30 min and was conducted as a brief introduction to the instrument followed by a group discussion of the different triggers in the instrument. The scenario duration was 14 min and 24 s and was divided in 1-min intervals, altogether 15. The ISRI score was calculated for each interval and rater as the difference between positive and negative trigger strengths. The IRR among the six raters was assessed using a two-way mixed, consistency, average-measures intra-class correlation (ICC) [26]. An ICC value over 0.60 is considered a good IRR [26].

#### Phase 4: comparison between ISRI and postquestionnaire outcomes

In an effort to test for convergent validity, ISRI was applied to all the 24 video-recorded scenarios and the outcome was compared with outcome from a postquestionnaire immersion instrument used during the same experiment. We expected the immersion to be higher in the contextualized condition.

The experiment had a randomized controlled crossover design. After each scenario (basic/contextualized), each participant completed an immersion postquestionnaire consisting of nine questions. The questions were derived from the 31-item instrument for measuring immersion presented by Jennett et al. [12], using a 5-point Likert scale from 1 (agree to a very low degree) to 5 (agree to a very high degree). The median of all responses to the questionnaire is used as the total immersion score. The rationale for adapting only a subset of the questions from the original questionnaire was that many of the original questions specifically refer to being immersed *in a digital game* and thus were not suitable or would be confusing in a live role-playing situation. We selected questions relevant to the simulation situation and removed (rather than altered) questions which were highly associated with digital games, e.g. “To what extent was your sense of being in the game environment stronger than your sense of being in the real world?” and “At any point did you find yourself become so involved that you were unaware you were even using controls?”

The ISRI score difference between the basic and contextualized scenarios was compared to the difference as measured by the questionnaire. It should be noted that the ISRI score was measured on team level (*n* = 12) with two ambulance nurses in each team while the questionnaire was applied on individual level (*n* = 24). Furthermore, the postquestionnaire captures the self-reported, subjective experience of participants while ISRI instrument is focused on immersion indicators. Finally, the ISRI instrument covers the whole chain of events in a scenario, whereas the questionnaire only will provide participants’ summative experiences. In all, these differences make a correlation between the two instruments difficult.

### Statistical analysis

A paired *t* test was used to analyse the differences in ISRI scores between the basic condition and the contextualized condition. To compare the participants’ postquestionnaire responses in the two conditions, a related-samples Wilcoxon signed rank test was used. A *p* value of <0.05 was considered significant in all statistical tests. For all other data, descriptive statistics were used. All statistical analyses were performed using the statistical software program SPSS 21.0 (SPSS Inc., Chicago, IL).

## Results

### Delphi process and activities

The panel consisted of 10 members, and the response rate from these panel members was 100 % in all four rounds.

As described in Table 2, the process started with panel members identifying 227 events that they considered as signs of reduced or enhanced immersion (round 1). By a clustering process, those 227 events were combined into 47 clusters. These were then combined into 11 triggers including a total of 21 subheadings. The role of the subheadings is to further detail the meaning of a trigger.

**Table 2:** Instrument development process: activities and results

	Activity	Results
Round 1	Panel members (*n* = 10) screened video recordings from two simulation scenarios and identified events considered as signs of reduced or enhanced immersion. The identified events were sorted into a matrix and clustered in two steps.	The panel members (*n* = 10) identified 227 events that they consider as signs of reduced or enhanced immersion.
		By a clustering process, the events were combined in 47 clusters. The 47 clusters were then combined to 11 triggers with 21 subheadings.
Round 2	The 11 triggers were sent back to the panel members (*n* = 10). The panel members were asked to classify the triggers as important or not important and adding potential new triggers.	10 triggers were scored as important by more than 80 % of the participants in the panel, and one trigger was deleted together with its two subheadings. No trigger was added to the list.
Round 3	Panel members (*n* = 10) were asked to classify triggers as signs of enhanced or reduced immersion. They also scored the importance of each trigger on a Likert scale: 1 = not important, 2 = not very important, 3 = possibly important, 4 = important and 5 = extremely important.	The panel members (*n* = 10) independently classified seven triggers as signs of reduced immersion and three as signs of enhanced immersion. All 10 triggers were classified as 4 or higher in importance by more than 80 % of the panel members. Three subheadings to the triggers were also added.
	The panel members were also asked about the wording of the triggers.	Four triggers got new wordings.
Round 4	A new scoring of the trigger importance.	All 10 triggers were classified as 4 or higher in importance by more than 80 % of the panel members.
Content validity analysis	The panel members (*n* = 10) were asked to individually grade the relevance of the 10 triggers using a 4-point rating scale to rate the relevance of each individual trigger: 1 = not relevant, 2 = somewhat relevant, 3 = quite relevant and 4 = highly relevant.	Trigger 6 reached an I-CVI = 0.8, triggers 2 and 10 I-CVI = 0.9 and the rest of the triggers I-CVI = 1.0.
		The S-CVI which measure the content validity of the overall scale reached an acceptable standard of S-CVI/Ave = 0.96.
Inter-rater reliability analysis	6 raters were independently rating the same video recording from a simulation. The scenario was divided in intervals. In each interval, the immersion value was calculated. The IRR was assessed using an intra-class correlation calculation.	The ICC for the ISRI score was 0.92.

In round 2, the panel members independently classified triggers as important or not important. Ten triggers were considered important by over 80 % of the panel members. In round 3, the panel members independently classified seven triggers as signs of reduced immersion and three as signs of enhanced immersion. All the 10 triggers were classified as 4 or higher in importance by over 80 % of the panel members. A new scoring of trigger importance in round 4 revealed that over 80 % of the panel members scored all 10 triggers as important (Table 2).

### Results from content validity analysis

All 10 of the original triggers met the acceptable I-CVIs standard. Trigger 6 reached an I-CVI = 0.8, triggers 2 and 10 I-CVI = 0.9 and the rest of the triggers I-CVI = 1.0. The S-CVI which measure the content validity of the overall scale reached an acceptable standard of S-CVI/Ave = 0.96 (Table 2)*.*

### The final instrument

ISRI, presented in Additional file 1, consists of 10 triggers (T1–T10) with 22 subheadings. A trigger is defined by a short sentence, and the subheadings give refinement and clarification. The triggers cover aspects related to intervention of the instructor (T1); problems using equipment (T2); jumps in time and/or space (T3); unnatural execution of operations (T4); unnatural interaction with the manikin or participants (T5); uncertainty of what is expected (T6); technological distractions (T7); natural responses to stimuli in the simulation (T8); natural interaction with the simulator (T9); and natural interaction with participants (T10). Triggers T1–T7 indicate reduced immersion and triggers T8–T10 indicate enhanced immersion.

When using the tool, a rater watches a video recording of a training scenario. When a situation arises that may be a sign of reduced or enhanced immersion, the rater stops the video and selects an appropriate trigger, optionally including a subheading. For each assigned trigger, the rater indicates the strength from 1 (weak indication) to 3 (strong indication). Raters are free to identify as many triggers as they find appropriate. It should be noted that a situation could include triggers that indicate enhanced as well as reduced immersion. The rater repeats this until the end of the scenario. The outcome of applying the instrument (being either a paper-based or digital protocol) is a list of triggers consisting of the scenario time, trigger number and strength.

### Results from the inter-rater reliability analysis

**Fig. 1:**
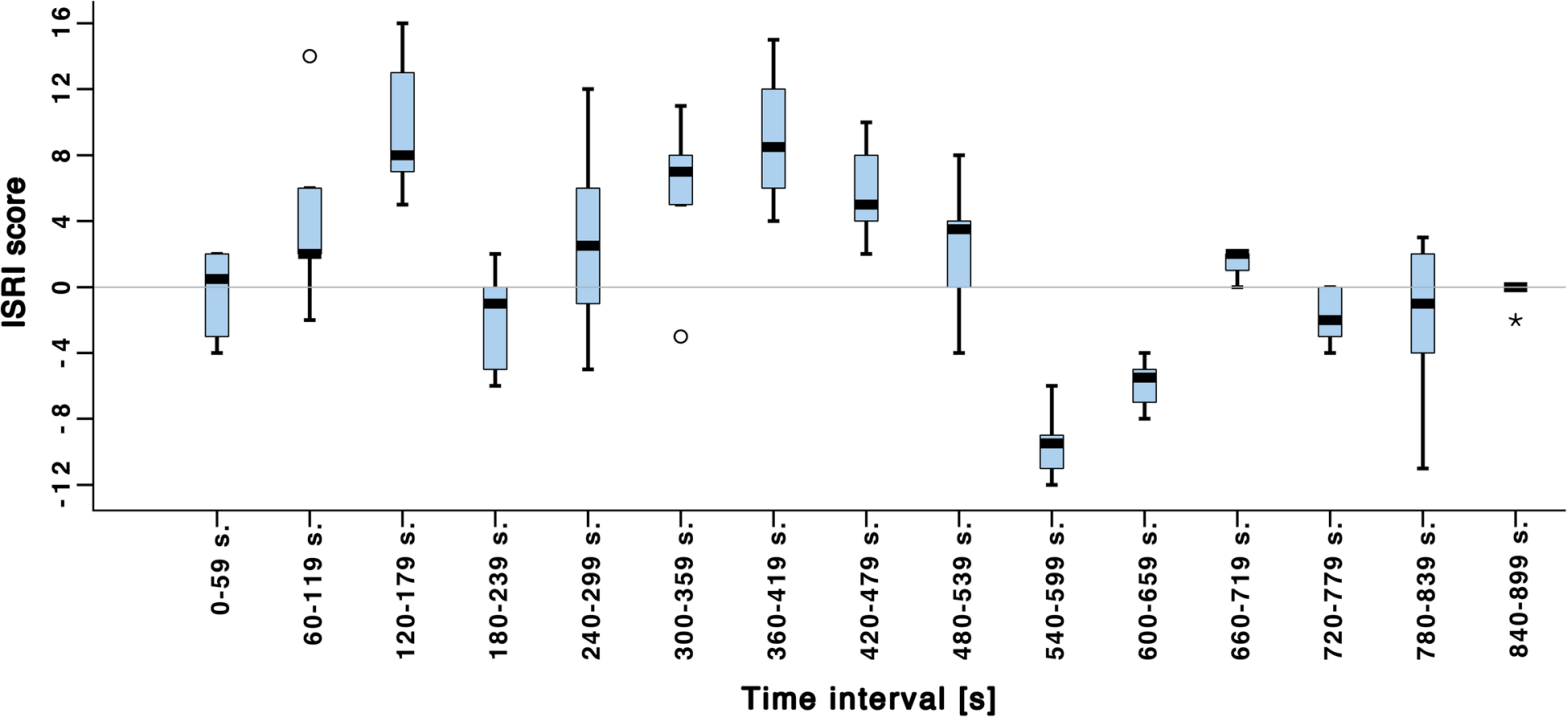
Boxplots of the ISRI scores from raters (*n* = 6) for each minute (*n* = 15) of the rated session. Potential outliers are marked with an *asterisk* (*) (>3.0 IQR from the box) and an *open circle* (o) (>1.5, ≤3 IQR from the box)

The ICC calculation based on the rating of one video recording showed an excellent agreement among the six raters. The ICC for the ISRI scores was 0.92 which indicates that the instrument is suitable for use to determine immersion among participants in a simulation scenario (Table 2 and Fig. 1).

As illustrated in Fig. 1, the ISRI score from the raters (*n* = 6) for each minute (*n* = 15) of the rated session was calculated. The ISRI score is computed by subtracting the sum of strengths assigned to triggers indicating reduced immersion (T1–T7) from the sum of strengths assigned to triggers indicating enhanced immersion (T8–T10). A positive ISRI score indicates that a rater had more strength assigned to triggers indicating enhanced immersion in an interval, compared to those assigned to triggers indicating reduced immersion. For example, Fig. 1 shows that all the raters agreed that the interval 120–179 s has a positive ISRI score while the interval 540–599 s has a negative ISRI score.

### Agreement between ISRI and a postquestionnaire

**Fig. 2:**
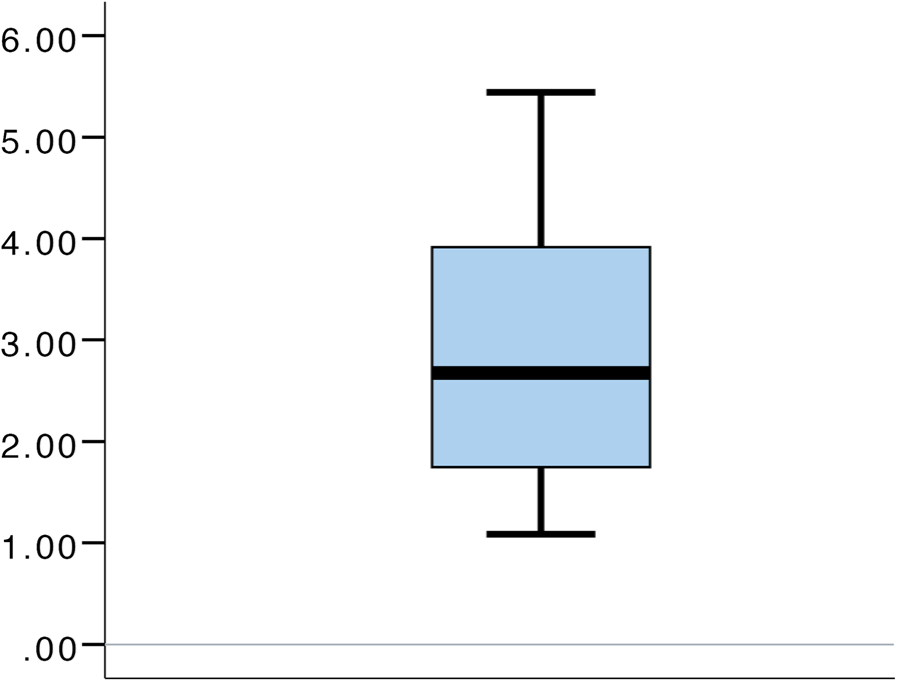
The boxplot shows the difference in ISRI score for teams (*n* = 12)

**Fig. 3:**
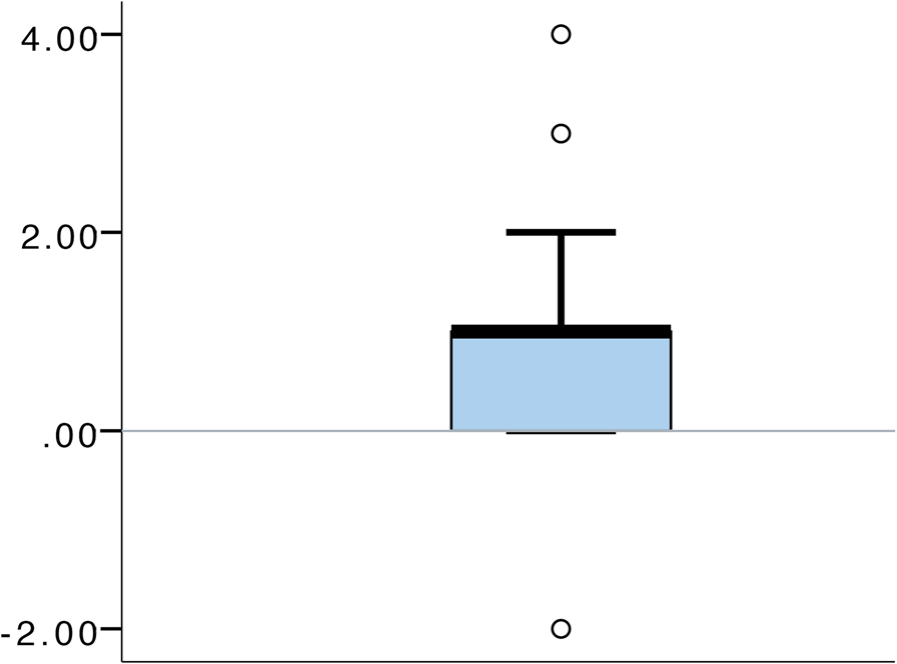
The boxplot shows difference in questionnaire score for individual subjects (*n* = 24). Potential outliers are marked with *open circles* (o) (>1.5, ≤3 IQR from the box)

A comparison between the ISRI results and the results from a questionnaire based on an established instrument suggests an agreement between the two measures. The mean ISRI score was 2.17 (sd = 1.67) in the contextualized condition and −0.77 (sd = 2.01) in the basic condition (*n* = 12). The mean within team difference was 2.94 (sd = 1.45). This difference is significant at *p* < 0.001, using a paired *t* test. This tendency is mirrored in participants’ postquestionnaire responses, exhibiting a median difference of 1.0 (*n* = 24). A related-samples Wilcoxon signed rank test shows these results to be significant at *p =* 0.005. Figure 2 shows a boxplot of the difference in ISRI score (*n* = 12). Figure 3 shows a boxplot of the differences in immersion postquestionnaire score (*n* = 24).

With ISRI (Fig. 2), all the teams exhibited higher ISRI score in the contextualized condition. As illustrated in Fig. 3, all the participants except one reported equal or higher immersion in the contextualized condition.

We expected increased immersion in the contextualized condition, which is supported by the postquestionnaire data, as well as the application of the ISRI instrument. Both detect a significantly higher immersion in the contextualized condition. As discussed above, it should be noted that it is not possible to do a direct comparison between the outcomes of these two instruments as they, among other things, are using different scales and have different granularities.

## Discussion

### Summary of results

We have developed a non-intrusive content-valid instrument for identifying and exploring immersion among participants in healthcare simulation scenarios. The results from the Delphi rounds and the developed instrument presented in this paper are in accordance with results from previous studies [27, 28] of how participants perceive a medical simulation. For example, role-playing is an important factor in several ISRI triggers (e.g. triggers 5 and 9), and role-playing has been described as an important factor for immersion in general [29] as well as in medical training [27]. One trigger in ISRI relates to participants’ uncertainty in what is expected or can be done in the simulated scenario. This trigger is related to illogical jumps in space and time and also to the trigger describing when all or parts of a task have to be pretended. Those were all identified as distinctive negative triggers. These triggers are similar to the findings in previous reports [26, 27]. For example, Dieckmann et al. [27] report that participants expressed hesitation and uncertainty of what was expected of them in terms of real patient care actions when interacting with a manikin. Horcik et al. [28] categorize participants’ concerns as being *close to the targeted work* or *close to the simulated work*. A constant presence of the latter type of concern led the authors to conclude that a stable immersion could not be observed in their study. This distinction between targeted and simulated work relates well to our distinction between enhanced and reduced immersion.

We argue that immersion is an important factor for healthcare simulation as it relates to what degree participants are involved in the fictional training scenario. There are many related terms used in the literature, such as *engagement*, *as-if* or *suspension of disbelief*, which are closely related to immersion. Furthermore, there is an ongoing discussion [30] regarding how fidelity of a simulation relates to the learning outcome. In this context, many authors emphasize the importance to focus on learners’ experience and engagement rather than the characteristics or properties of the physical equipment [27, 31, 32]. We argue, in line with, e.g. Hamstra et al. [31], that emphasis should be placed on the relation between educational effectiveness and engagement rather than the physical resemblance of the simulator equipment. Hence, the fidelity (functional and structural) of a simulation can be regarded as a mediator for immersion and learning. Conceptually, the relation between these aspects is not well understood. ISRI has the potential to be used in future studies of what this connection entails and how it works, e.g. how different scenario designs or simulation environments affect future performance and learning.

### Implications

There are some practical advantages of using ISRI in comparison to questionnaire instruments. Firstly, ISRI allows non-intrusive grading with minimal effect on study participants. Secondly, it allows several graders and thus high-quality grading. Thirdly, when applied to video recordings, analysis can be done independent of time and place in relation to when a simulation or experiment is taking place. We do not, however, see any problems using ISRI in live situations, beyond the obvious risks/effects of observer presence. Fourthly, in addition to providing a quantitative outcome score, ISRI provides opportunities to do a richer analysis of individual trigger types and their occurrences. In particular, in the computer-based version of the instrument, all occurrences of a specific trigger type can be extracted from the video recordings, allowing an analysis of their occurrences during different phases of a simulated scenario.

As suggested by Dede [4], immersive environments need to be designed and adapted to specific disciplines and subject areas. Here, ISRI can be of practical use to identify situations that have positive or negative effects on immersion. This knowledge can help simulation educators to decide the optimal level of different fidelity dimensions [20] to create adequate and engaging learning environments.

### Limitations

A limitation of the presented work is related to the study design and the selection of the 10 members in the expert panel. A different group of experts could possibly lead to different contents of the instrument. Furthermore, the six participants in the inter-rater measurements were all part of the expert panel. This could be a potential problem.

When developing the instrument we used a computer-based instrument version that was integrated within a computer program containing the video recordings of the simulation scenarios. A rater examined the video recording, and when a trigger was identified, the rater could stop the video and score the sequence directly in the program. All scoring and times could then be extracted as raw data. Hence, the paper-based instrument appended to this paper was not used during the development process. It is possible that this may affect the practical use of the instrument. On the other hand, the instrument is exactly the same; it is only the format that differs.

The IRR validation presented in this paper is for the overall ISRI score. The choice of individual trigger types has not been formally validated. We suggest that the use of the instrument is preceded by inter-rater training. In the present study, an inter-rater training session was performed before the IRR analysis. The training session lasted 30 min and was conducted as a brief introduction to the instrument and a group discussion of the different triggers in the instrument. Rater bias is a well-known problem in all rating, and inter-rater training is an effective and common method to reduce this problem [24]. It is possible that an inter-rater training will influence the IRR analysis, but on the other hand, as recommended by Hasson et al. [24], inter-rater training should precede all scoring including multiple raters. Finally, it should be noted that the comparison between ISRI and Jennett et al.’s immersion questionnaire has limitations with respect to scale as well as granularity.

## Conclusions

We have developed a content-valid instrument for rating and identifying immersion among participants in healthcare simulation scenarios. The instrument can be used as a tool to further research the relation between immersion and fidelity, which in turn may have consequences for learning. Our instrument may also be used to analyse and improve simulation models and scenario design of healthcare personnel.

## Additional file

Additional file 1: Immersion Score Rating Instrument.
