# Supplementary material for: Measuring participants’ immersion in healthcare simulation: the development of an instrument
Source: Adv Simul (Lond). 2016 May 23;1:17. doi: 10.1186/s41077-016-0018-x (PMC5806227; doi:10.1186/s41077-016-0018-x)
Supplement: Supplementary file 1 — Immersion Score Rating Instrument. [file 41077_2016_18_MOESM1_ESM.docx]

# Immersion Score Rating Instrument

Version 1.0

## Triggers

**1: Destructive interaction between participants and persons outside the scenario**

1:1 A participant asks the instructor for directions

1:2 An instructor asks questions or gives instructions

- Strong indication of reduced immersion = 3
- Neutral indication of reduced immersion = 2
- Weak indication of reduced immersion = 1

**2: A participant expresses that the expected equipment is missing or not functioning normally**

2:1 Expected equipment is missing

2:2 A participant has trouble using the existing equipment

- Strong indication of reduced immersion = 3
- Neutral indication of reduced immersion = 2
- Weak indication of reduced immersion = 1

**3: Disturbing jumps in time and/or space**

3:1 A jump initiated by an instructor

3:2 A jump initiated by a participant

- Strong indication of reduced immersion = 3
- Neutral indication of reduced immersion = 2
- Weak indication of reduced immersion = 1

**4: All or part of the operations are pretended**

4:1 An instructor asks a participant to pretend a moment

4:2 A participant claims to perform one operation without doing it

- Strong indication of reduced immersion = 3
- Neutral indication of reduced immersion = 2
- Weak indication of reduced immersion = 1

**5: Unnatural interaction with the patient and/or another person in the scenario**

5:1 A participant acts or communicates in a way that would be unnatural towards a patient in a natural context

5:2 A participant acts or communicates in a way that would be unnatural towards another person in a natural context

- Strong indication of reduced immersion = 3
- Neutral indication of reduced immersion = 2
- Weak indication of reduced immersion = 1

**6: Uncertainty in what is expected or can be done in the simulated scenario**

6:1 Participants discuss technical aspects of the scenario

6:2 A participant expresses uncertainty about what is expected or can be done in the scenario

- Strong indication of reduced immersion = 3
- Neutral indication of reduced immersion = 2
- Weak indication of reduced immersion = 1

**7: Technology that would not be part of a natural context disturbs the participants**

7:1 A simulator does not work as intended

7:2 A camera/microphone disturbs a participant

7:3 Other equipment that does not belong to the scenario disturbs a participant

- Strong indication of reduced immersion = 3
- Neutral indication of reduced immersion = 2
- Weak indication of reduced immersion = 1

**8: Natural responses to stimuli in the scenario**

8:1 A participant responds to something non-patient-related

8:2 A participant responds to medical findings

- Strong indication of enhanced immersion = 3
- Neutral indication of enhanced immersion = 2
- Weak indication of enhanced immersion = 1

**9: Natural interaction with the simulator**

9:1 A participant informs the simulator

9:2 A participant performs actions or investigations that a simulator can not provide answers to

9:3 A participant asks the simulator to perform an action that is not possible (e.g. ask the simulator to rise)

- Strong indication of enhanced immersion = 3
- Neutral indication of enhanced immersion = 2
- Weak indication of enhanced immersion = 1

**10: Natural interaction and/or verbal communication with another person in the scenario**

10:1 Natural interaction and/or verbal communication within the team and with other health care professionals

10:2 Natural interaction and/or verbal communication with relatives or bystanders

- Strong indication of enhanced immersion = 3
- Neutral indication of enhanced immersion = 2
- Weak indication of enhanced immersion = 1

## Instructions

The purpose of this instrument is to identify signs in a simulation scenario, which indicate a reduced or enhanced immersion of scenario participants. Trigger numbers 1-7 are negative, indicating reduced immersion and trigger numbers 8-10 are positive, indicating enhanced immersion. For every trigger, there are one or more subheadings. These serve to define situations and to provide guidance for the reviewer.

*Implementation:*

1) Watch the video recording from the start of the scenario (time = 0)

2) Stop the video recording when a situation arises that may be a sign of reduced or enhanced immersion of the participants. Note the scenario time.

3) Locate the appropriate trigger number (1-10)

4) Locate the appropriate subheading and compare with the situation where the video was stopped. If an appropriate subheading is missing, only use main trigger

5) Rate the strength of the situation on a 3-point scale: 1 = weak indication, 2 = neutral indication, 3 = strong indication

6) Start the video recording again and continue the evaluation until the end of the scenario

**Protocol: Immersion Score Rating Instrument**

Rater:……………………………………….

Scenario:……………………………………

| **Scenario time** | **Trigger number** | **Strength (1-3)** |
| --- | --- | --- |
|  |  |  |
|  |  |  |
|  |  |  |
|  |  |  |
|  |  |  |
|  |  |  |
|  |  |  |
|  |  |  |
|  |  |  |
|  |  |  |
|  |  |  |
|  |  |  |
|  |  |  |
|  |  |  |
|  |  |  |
|  |  |  |
|  |  |  |
|  |  |  |
|  |  |  |
|  |  |  |
|  |  |  |
|  |  |  |
|  |  |  |
|  |  |  |
|  |  |  |
|  |  |  |
|  |  |  |
|  |  |  |
|  |  |  |
|  |  |  |
|  |  |  |
|  |  |  |
|  |  |  |
|  |  |  |
